# Identification of key predictors of postmenopausal osteoporosis from routine clinical indicators using explainable machine learning

**DOI:** 10.1371/journal.pone.0351334

**Published:** 2026-06-24

**Authors:** Yang Guo, Shuai Jiang, Wei Zhu, Longwang Tan, Chuang Liu, Yongjun Jia, Chi Zhang, Kok-Yong Chin

**Affiliations:** 1 Department of Nursing, School of Medicine, Shaanxi Institute of International Trade & Commerce, Xianyang, Shaanxi, China; 2 Medical Morphology Experiment Centre, School of Basic Medicine, Hunan University of Medicine, Huaihua, Hunan, China; 3 Department of Orthopaedics, Affiliated Hospital of Xizang Mizu University, Xianyang, Shaanxi, China; 4 Division of Spinal Surgery, Department of Nursing, Affiliated Hospital of Shaanxi University of Chinese Medicine, Xianyang, Shaanxi, China; 5 Rehabilitation Department (Area 8), Xi’an Daxing Hospital, Xi’an, Shaanxi, China; 6 Department of Pharmacology, Faculty of Medicine, University Kebangsaan Malaysia, Cheras, Malaysia; University of Padova: Universita degli Studi di Padova, ITALY

## Abstract

**Background:**

Machine learning (ML) shows promise in using clinical data to predict chronic diseases. However, its application in PMOP risk assessment using readily available clinical and biochemical parameters is underexplored.

**Objective:**

This study aimed to develop and validate an interpretable ML-based model for assessing PMOP using clinical features and laboratory biomarkers, and to identify factors associated with PMOP using SHapley Additive exPlanations (SHAP).

**Methods:**

A retrospective cross-sectional study included 1,717 postmenopausal women from two hospitals in Northwest China. PMOP was diagnosed with dual-energy X-ray absorptiometry (DXA T-score ≤−2.5). Data collected included demographics, clinical details, and various laboratory parameters, such as bone metabolism markers, 25-hydroxyvitamin D [25-(OH)D], electrolytes, and routine blood counts. Ten ML algorithms were employed for feature selection and model construction on a dataset split into training (n = 1201) and testing (n = 516) sets. Performance was evaluated using the Area Under the receiver operating characteristic curve (AUC), accuracy, sensitivity, specificity, and calibration.

**Results:**

The Extra Trees (ET) model achieved the best test-set performance, with an AUC of 0.717 (95% CI: 0.682–0.752). SHAP analysis revealed that age was the most significant associated factor (SHAP value: 0.0648), followed by body mass index (BMI) (0.0243) and chloride ion levels (0.0209). Other top predictors included the use of antihypertensive drugs and years since menopause.

**Conclusion:**

The ET ML algorithm showed the best performance in assessing PMOP, with age, BMI, and circulating chloride levels as significant associated factors.

## Introduction

Osteoporosis is a systemic metabolic disorder characterized by an imbalance in bone homeostasis, reduced bone mass, and deterioration of bone microarchitecture, leading to increased bone fragility and susceptibility to fractures [1,2]. It exhibits a high global prevalence, ranking as the third most common chronic disease following cardiovascular diseases and diabetes [3]. Postmenopausal osteoporosis (PMOP), the most common type of primary osteoporosis, is triggered by the decline in ovarian function after menopause [4]. In China, the prevalence of osteoporosis is substantially higher in women than in men, particularly among individuals aged ≥50 years (19.2%), and reaches 51.6% in women over 65 years [5–7]. Most patients show no obvious symptoms in the early stages, but as the disease progresses, clinical manifestations, such as chronic low back pain, fatigue, and loss of height, may gradually appear [5].

With increasing clinical and research focus on osteoporosis-related diseases, a growing number of bone turnover markers relevant to the auxiliary diagnosis and treatment of osteoporosis are being explored. Bone turnover markers play a crucial role in early screening and therapeutic monitoring of osteoporosis, offering high sensitivity and specificity [8,9]. Beyond traditional bone turnover markers, electrolyte levels are recognized as significant factors influencing bone metabolism [10]. Electrolytes, such as calcium, phosphorus, magnesium, sodium, and potassium, play crucial roles in maintaining a balance of bone mineralization, bone remodeling, and cellular function [11]. Their dysregulation is closely associated with the onset and progression of osteoporosis [12].

Among these, serum calcium, as the primary mineral component of bone, directly influences the dynamic balance between bone formation and resorption by altering its concentration, serving as a key indicator of bone metabolic status [13]. For instance, calcium ions directly influence osteoblast and osteoclast activity via the calcium-sensing receptor signaling pathway [14,15]. Serum phosphorus works synergistically with calcium in the process of bone salt deposition. An imbalance in their ratio can lead to impaired bone mineralization. Serum magnesium indirectly affects bone density and strength by regulating parathyroid hormone (PTH) and vitamin D metabolism [16]. Chloride ions (CI^-^) participate in osteoclast-mediated bone resorption through Anoctamin-1 (ANO1) CI^-^ channels [17]. Furthermore, sodium and potassium ions also play regulatory roles in bone metabolism. A high-sodium diet can promote urinary calcium excretion, leading to bone calcium loss [18]. In contrast, adequate potassium intake helps buffer the acid load and reduce calcium loss, thereby exerting a protective effect on bone [19]. Therefore, electrolyte levels not only reflect the body’s metabolic homeostasis but may also serve as important indicators of osteoporosis risk and guide clinical interventions.

Vitamin D and PTH are critical regulators of calcium-phosphorus metabolism and bone mineralization. The most reliable biomarker of vitamin D status is serum 25-hydroxyvitamin D [25(OH)D], which maintains calcium and phosphorus balance by promoting their intestinal absorption, renal reabsorption, and bone deposition, thereby ensuring bone health and mechanical strength [20]. Insufficient vitamin D is closely associated with reduced bone mass and increased adiposity [21]. PTH, secreted by the parathyroid glands, regulates bone remodeling through its dual anabolic and catabolic effects depending on the secretion pattern and concentration. Clinically, monitoring vitamin D and PTH levels provides important guidance for diagnosing metabolic bone disorders and optimizing the management of osteoporosis [20,21].

With the rapid development of artificial intelligence (AI) and its applications in clinical research, technologies such as machine learning (ML) and deep learning (DL) can extract clinical information from large datasets to aid clinical decision-making [22]. Many ML techniques have been used to develop chronic disease prediction models, most of which show good predictive performance [23,24]. Although several ML models have been proposed for osteoporosis risk prediction, most lack interpretability, rely on specialized biomarkers, or have not been validated in community-dwelling postmenopausal populations. Therefore, this study aims to develop an explainable ML model using only routine clinical indicators to assess osteoporosis risk in postmenopausal women; identify key predictors using SHAP analysis; fill the gap between model performance and clinical interpretability, and evaluate their value in identifying osteoporosis. This study is expected to provide a reference for the adoption and integration of ML technology in bone health management.

## Materials and methods

### Study design and subjects

This retrospective study included postmenopausal patients hospitalized in the orthopedic wards of two tertiary hospitals in northwest China between 01/01/2024 and 30/06/2025. Clinical data were extracted from electronic medical records. The study protocol was reviewed and approved by the Ethics Committee of the School of Medicine, Shaanxi Institute of International Commerce & Trade (Approval No.: YYXY-HLX-2024-12-25) on December 25, 2024. The researchers accessed the fully anonymized data for analysis between 01/07/2025 and 31/08/2025. Thus, all data collection (i.e., data extraction) was performed after ethical approval, and patient consent was waived due to the retrospective and anonymized nature of the study.

Subjects were screened based on the following inclusion and exclusion criteria. Participants were eligible for inclusion if they were menopausal women aged 50 years or older, had complete body mass index (BMI), imaging, and laboratory data, had not received prior osteoporosis treatment, and had a diagnosis of primary osteoporosis. Individuals were excluded if they had other metabolic bone diseases (e.g., osteomalacia, Paget disease, or rickets), medical conditions associated with secondary osteoporosis (including hyperthyroidism, Cushing syndrome, hyperprolactinemia, hematological disorders, connective tissue diseases such as rheumatoid arthritis and systemic lupus erythematosus, bone tumors, or chronic kidney failure), were taking medications known to affect bone metabolism (e.g., hormone replacement or ablation therapy, glucocorticoids, thyroid supplements, anticonvulsants, warfarin, thiazide diuretics, or chemotherapy agents), had metal implants that could interfere with dual-energy X-ray absorptiometry (DXA) scans, or had incomplete clinical data.

### Diagnosis of osteoporosis

The diagnosis of osteoporosis was based on the lumbar spine (L1-L4) BMD, which was determined by DXA, as defined by the World Health Organization (WHO) in 1994. Based on BMD values, T-scores for subjects were calculated and categorized as in Table 1.

**Table 1:** Diagnostic criteria for osteoporosis.

Category	T-score cut-off value
Normal bone mass	≥ −1.0
Low bone mass or reduced bone mass	−2.5 to −1.0
Osteoporosis	≤ −2.5
Severe osteoporosis	≤ −2.5, accompanied by one or more fractures

### Data collection

The cases that met the requirements from 01/01/2024 to 30/06/2025 were screened and included. All patient data were fully anonymized prior to researcher access, and the study protocol was reviewed and approved by the Institutional Review Board of the participating institutions. The ethics committee waived the requirement for informed consent due to the retrospective and fully anonymized nature of the research. The data retrieved from the electronic medical record management systems of the two hospitals included the patient’s age, height, weight, BMI, past medical history (including medications used and surgical history). Data of patients’ electrolytes (Hitachi 7600, Hitachi High-Tech, Tokyo, Japan) were also retrieved; bone metabolism markers (MAGLUMI-X6, Shenzhen New Industry Biomedical Engineering Co., Ltd., Shenzhen, China), 25-OH-D (Abbott A3600, Abbott Diagnostics, Illinois, USA), blood routine (Murray BC-5309, Shenzhen Mindray Bio-Medical Electronics Co., Ltd, Shenzhen, China). Both hospitals used MEDIX-DR (Medilink/DMS Imaging, le Montougeux, France) to assess BMD at L1-L4. The patients’ BMD and T-scores were retrieved from the electronic medical record. If the record was unavailable, the hospital’s imaging department was contacted to identify the records through the patient’s name or medical record number.

### Feature selection

In this study, a systematic and comprehensive feature screening was conducted to identify key factors associated with osteoporosis in postmenopausal women. Specifically, we combined 10 distinct machine learning algorithms to ensure robustness and comprehensive feature selection. These algorithms included: Logistic Regression (LR, class weight = balanced), SVM-RBF (SVM), Decision Tree (DT), Random Forest (RF), Extra Trees (ET), Gradient Boosting (GB), AdaBoost (ADA), XGBoost (XGB), K-Nearest Neighbors (KNN), and Naive Bayes (NB). During the feature selection process, each algorithm employed the Recursive Feature Elimination (RFE) method, which iteratively removes less informative variables based on assessment performance to optimize model performance. The study sample was randomly split into a training set (n = 1201) and a test set (n = 516) at a ratio of 7:3 [25]. During the training phase, we further applied 10-fold cross-validation to evaluate model performance and stability [26]. Although all 30 features were retained based on RFE performance, multicollinearity and overfitting remain potential concerns. To mitigate these issues, we applied 10-fold cross-validation during training and used SHAP analysis post hoc to evaluate feature contributions, thereby identifying redundant variables for future simplification.

### Model verification

In this study, the best-performing model was evaluated using internal validation on a hold-out test set. The Youden index was employed to determine the optimal threshold, as it maximizes the combined benefit of sensitivity and specificity. During validation, a cumulative lift chart was used to assess the model’s performance and compare it with random selection. A confusion matrix was constructed to visually illustrate the discrepancies between assessment outcomes and actual observations. Model performance was evaluated using metrics including accuracy, sensitivity, specificity, and the area under the receiver operating characteristic curve (AUC-ROC). Furthermore, model calibration was examined by comparing assessment probabilities with actual observed outcomes.

### Characteristic importance assessment

SHAP values were used to measure the importance and contribution of each input variable to the model output [27]. As an interpretation method based on game theory, SHAP extends the classical Shapley value to interpret outcomes from machine learning models. By reasonably allocating the “contribution share” of features to assessments, it achieves optimal attribution for model outputs. This method enables researchers to gain deeper insights into the relative influence of various factors on osteoporosis risk assessment, thereby enhancing the model’s interpretability and clinical application value.

### Statistical analysis

Statistical analysis using SPSS 26.0 (IBM, Armonk, NY, USA) and Python 3.10.1 (Python Software Foundation) software. Classification variables were expressed as frequencies or proportions and compared using the chi-square test or Fisher’s exact test. The Kolmogorov-Smirnov-Lilliefors (K-S-L) test was used to test the normality of continuous data. Non-normal distribution variables were evaluated using the Wilcoxon rank sum test and are presented as median, first quartile (Q1), and third quartile (Q3). When P < 0.05, the difference was considered significant.

## Results

### Baseline data evaluation

This study enrolled postmenopausal female patients from the orthopedic wards of two tertiary Grade A hospitals in Northwest China. A total of 1717 cases were included based on the predetermined inclusion and exclusion criteria. Among them, 819 individuals (47.70%) were diagnosed with osteoporosis, while 898 (52.30%) were not. All clinical data were obtained from patients’ electronic medical records, totaling 30 clinical features. Detailed characteristics are presented in Table 2. The cohort was randomly divided into a training set (n = 1201) and a test set (n = 516) at a 7:3 ratio, using stratified sampling. There were no statistically significant differences in the distributions of variables between the two subsets, as shown in Table 3.

**Table 2:** Baseline characteristics of patients with OP.

Variable	Overall (1717)	No-OP (898)	OP (819)	Statistics	P-value
Age (year)	67.86 ± 8.44	65.63 ± 8.05	70.31 ± 8.19	250252	<0.001
BMI	23.62 ± 3.40	24.17 ± 3.31	23.01 ± 3.39	438781	<0.001
25-(OH)D (ng/mL)	36.80 ± 19.28	36.76 ± 18.82	36.85 ± 19.78	371644	0.703
N-MID (ng/mL)	17.86 ± 13.75	17.27 ± 14.69	18.51 ± 12.62	327385	<0.0001
P1NP (ng/mL)	62.85 ± 29.39	62.00 ± 26.21	63.79 ± 32.51	367287.5	0.9656
BALP (μg/L)	100.12 ± 19.40	98.73 ± 19.25	101.64 ± 19.47	336695	0.0013
β-CTX (ng/mL)	1.12 ± 19.08	1.51 ± 26.38	0.68 ± 0.37	348620	0.0625
WBC (10^9^/L)	5.54 ± 1.97	5.57 ± 1.92	5.52 ± 2.03	378469	0.2954
NEUT (%)	60.28 ± 11.50	59.21 ± 11.34	61.45 ± 11.55	320316.5	<0.0001
RBC (10^12^/L)	4.24 ± 2.86	4.27 ± 1.41	4.21 ± 3.87	452079.5	<0.0001
HBG (g/L)	123.92 ± 15.80	126.32 ± 14.33	121.29 ± 16.89	439992	<0.0001
HCT (%)	38.53 ± 5.38	39.16 ± 4.89	37.83 ± 5.81	436506.5	<0.0001
PC (g/L)	199.53 ± 65.85	203.54 ± 62.03	195.13 ± 69.56	405968.5	0.0002
TP (g/L)	66.75 ± 15.31	66.70 ± 6.17	66.81 ± 21.22	385973.5	0.0754
ALB (g/L)	39.48 ± 4.38	40.16 ± 4.06	38.75 ± 4.59	432790.5	<0.0001
GLB (g/L)	26.88 ± 4.87	26.51 ± 4.59	27.29 ± 5.13	331606	0.0004
A/G	1.52 ± 0.33	1.57 ± 0.32	1.47 ± 0.34	432991	<0.0001
GLU (mmol/L)	5.44 ± 1.51	5.50 ± 1.40	5.38 ± 1.61	401133.5	0.0011
K^+^ (mmol/L)	4.02 ± 0.39	4.02 ± 0.37	4.01 ± 0.40	372816	0.6201
Na^+^ (mmol/L)	142.39 ± 3.89	142.74 ± 4.56	142.00 ± 2.95	427610.5	<0.0001
CL^-^ (mmol/L)	106.21 ± 4.51	105.79 ± 4.72	106.68 ± 4.22	295528	<0.0001
Ca^2+^ (mmol/L)	2.27 ± 0.15	2.29 ± 0.14	2.26 ± 0.16	422827.5	<0.0001
Years since menopause (year)	17.86 ± 8.44	15.63 ± 8.05	20.31 ± 8.19	240248	<0.001
Smoking					
Yes	3 (0.2%)	3 (0.3%)	0 (0.0%)	0.442	0.251
No	1714 (99.8%)	895 (99.7%)	819 (100.0%)		
History of surgery					
Yes	1057 (61.6%)	547 (60.9%)	510 (62.3%)	0.334	0.563
No	660 (38.4%)	351 (39.1%)	309 (37.7%)		
Use of antihypertensive drugs					
Yes	1695 (98.7%)	887 (98.8%)	808 (98.7%)	0.047	0.828
No	22 (1.3%)	11 (1.2%)	11 (1.3%)		
Use of hypoglycemic drugs					
Yes	489 (28.5%)	276 (30.7%)	213 (26.0%)	4.7	0.030
No	1228 (71.5%)	622 (69.3%)	609 (74.0%)		
Use of lipid-lowering drugs					
Yes	144 (8.4%)	91 (10.1%)	53 (6.5%)	7.477	0.006
No	1573 (91.6%)	807 (89.9%)	766 (93.5%)		
Use of other drugs					
Yes	187 (10.9%)	113 (12.6%)	74 (9.0%)	5.556	0.018
No	1530 (89.1%)	785 (87.4%)	745 (91.0%)		
Presence of other diseases					
Yes	291 (16.9%)	149 (16.6%)	142 (17.3%)	0.169	0.681
No	1426 (83.1%)	749 (83.4%)	677 (82.7%)		

Among the data included in this study, all female participants had no alcohol consumption habit. Therefore, this variable was excluded from the baseline characteristics. Although this avoided missing data, it also prevented us from assessing alcohol as a potential confounder. Future studies should include diverse populations with varying alcohol intake to evaluate its role in PMOP. 25-(OH)D (25-Hydroxyvitamin D), N-MID (N-terminal mid-fragment of osteocalcin), P1NP (Procollagen type I N-terminal propeptide), BALP (Bone-specific Alkaline Phosphatase), β-CTX (β-Cross-linked C-telopeptide of type I collagen), WBC (White Blood Cell Count), NEUT% (Neutrophil Percentage), RBC (Red Blood Cell Count), HBG (Hemoglobin), HCT (Hematocrit), PC (Platelet Count), TP (Total Protein), ALB (Albumin), GLB(Globulin), A/G (Albumin/Globulin Ratio), GLU (Glucose), K^+^ (Potassium Ion), Na^+^ (Sodium Ion), CL^-^ (Chloride Ion), Ca^2+^ (Calcium Ion).

**Table 3:** Baseline characteristics of training set and test set.

Variable	Overall (1717)	No-OP (898)	OP (819)	Statistics	P-value
Age (year)	67.86 ± 8.44	67.77 ± 8.44	68.08 ± 8.45	302821	0.455
BMI (kg/cm^2^)	23.62 ± 3.40	23.64 ± 3.38	23.57 ± 3.42	315024	0.583
25-(OH)D (ng/mL)	36.80 ± 19.28	36.76 ± 18.82	36.85 ± 19.78	296775.5	0.1649
N-MID (ng/mL)	17.86 ± 13.75	17.27 ± 14.69	18.51 ± 12.62	290384.5	0.0387
P1NP (ng/mL)	62.85 ± 29.39	62.00 ± 26.21	63.79 ± 32.51	299593	0.2758
BALP (μg/L)	100.12 ± 19.40	98.73 ± 19.25	101.64 ± 19.47	295187	0.0986
β-CTX (ng/mL)	1.12 ± 19.08	1.51 ± 26.38	0.68 ± 0.37	307672.5	0.8166
WBC (10^9^/L)	5.54 ± 1.97	5.57 ± 1.92	5.52 ± 2.03	316414	0.4864
NEUT (%)	60.28 ± 11.50	59.21 ± 11.34	61.45 ± 11.55	307481	0.8008
RBC (10^12^/L)	4.24 ± 2.86	4.27 ± 1.41	4.21 ± 3.87	319270.5	0.3176
HBG (g/L)	123.92 ± 15.80	126.32 ± 14.33	121.29 ± 16.89	316543	0.4778
HCT (%)	38.53 ± 5.38	39.16 ± 4.89	37.83 ± 5.81	318026.5	0.3857
PC (g/L)	199.53 ± 65.85	203.54 ± 62.03	195.13 ± 69.56	315866.5	0.5236
TP (g/L)	66.75 ± 15.31	66.70 ± 6.17	66.81 ± 21.22	315164.5	0.5732
ALB (g/L)	39.48 ± 4.38	40.16 ± 4.06	38.75 ± 4.59	316334.5	0.4917
GLB (g/L)	26.88 ± 4.87	26.51 ± 4.59	27.29 ± 5.13	307683.5	0.8175
A/G	1.52 ± 0.33	1.57 ± 0.32	1.47 ± 0.34	317097	0.4418
GLU (mmol/L)	5.44 ± 1.51	5.50 ± 1.40	5.38 ± 1.61	312205	0.8033
K^+^ (mmol/L)	4.02 ± 0.39	4.02 ± 0.37	4.01 ± 0.40	307027	0.7637
Na^+^ (mmol/L)	142.39 ± 3.89	142.74 ± 4.56	142.00 ± 2.95	307713.5	0.8197
CL^-^ (mmol/L)	106.21 ± 4.51	105.79 ± 4.72	106.68 ± 4.22	293148	0.0759
Ca^2+^ (mmol/L)	2.27 ± 0.15	2.29 ± 0.14	2.26 ± 0.16	323614	0.1441
Years since menopause (year)	17.86 ± 8.44	17.77 ± 8.44	18.08 ± 8.45	302920	0.4550
Smoking					
Yes	3(0.2%)	2(0.2%)	1 (0.2%)	–	1.000
No	1714(99.8%)	1199(99.8%)	515(99.8%)		
History of surgery					
Yes	1057 (61.6%)	729(60.7%)	328 (63.6%)	1.253	0.263
No	660(38.4%)	472(39.3%)	188(36.4%)		
Use of antihypertensive drugs					
Yes	1695(98.7%)	1182(98.4%)	513(99.4%)	2.857	0.091
No	22(1.3%)	19(1.6%)	3(0.6%)		
Use of hypoglycemic drugs					
Yes	489 (28.5%)	333(27.7%)	156(30.2%)	1.113	0.292
No	1228(71.5%)	868(72.3%)	360(69.8%)		
Use of lipid-lowering drugs					
Yes	144(8.4%)	108(9.0%)	36(7.0%)	1.909	0.167
No	1573(91.6%)	1093(91.0%)	480(93.0%)		
Use of other drugs					
Yes	187 (10.9%)	117 (9.7%)	70(13.6%)	5.438	0.020
No	1530(89.1%)	1084(90.3%)	446(86.4%)		
Presence of other diseases					
Yes	291 (16.9%)	197(16.4%)	94(18.2%)	0.844	0.358
No	1426(83.1%)	1004(83.6%)	422(81.8%)		

Among the data included in this study, all female participants had no alcohol consumption habit. Therefore, this variable was excluded from the baseline characteristics. Although this avoided missing data, it also prevented us from assessing alcohol as a potential confounder. Future studies should include diverse populations with varying alcohol intake to evaluate its role in PMOP. 25-(OH)D (25-Hydroxyvitamin D), N-MID (N-terminal mid-fragment of osteocalcin), P1NP (Procollagen type I N-terminal propeptide), BALP (Bone-specific Alkaline Phosphatase), β-CTX (β-Cross-linked C-telopeptide of type I collagen), WBC (White Blood Cell Count), NEUT% (Neutrophil Percentage), RBC (Red Blood Cell Count), HBG (Hemoglobin), HCT (Hematocrit), PC (Platelet Count), TP (Total Protein), ALB (Albumin), GLB(Globulin), A/G (Albumin/Globulin Ratio), GLU (Glucose), K+ (Potassium Ion), Na+ (Sodium Ion), CL- (Chloride Ion), Ca2+ (Calcium Ion).

### Machine learning-based recursive prediction of OP

**Fig 1:**
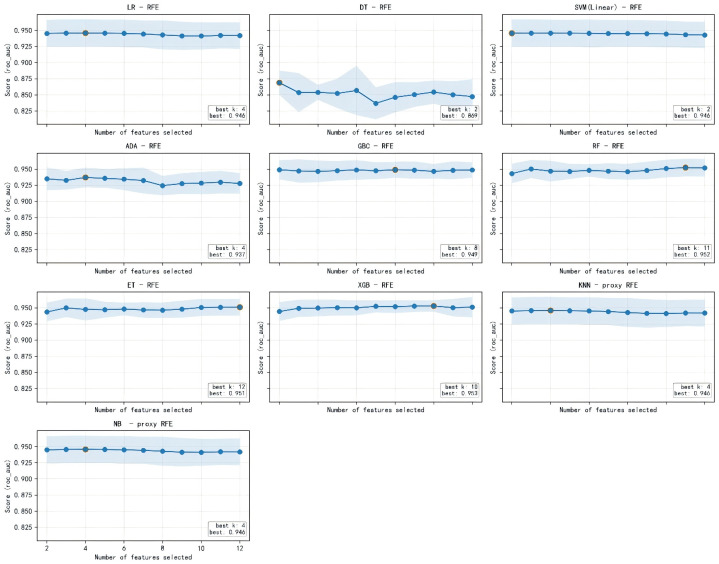
Results of 10 different algorithms using recursive feature elimination (RFE) procedure for feature selection. Logistic Regression (LR, class_weight = balanced), SVM-RBF (SVM), Decision Tree (DT), Random Forest (RF), Extra Trees (ET), Gradient Boosting (GB), AdaBoost (ADA), XGBoost (XGB), KNN (K-Nearest Neighbors), Naive Bayes (NB).

**Fig 2:**
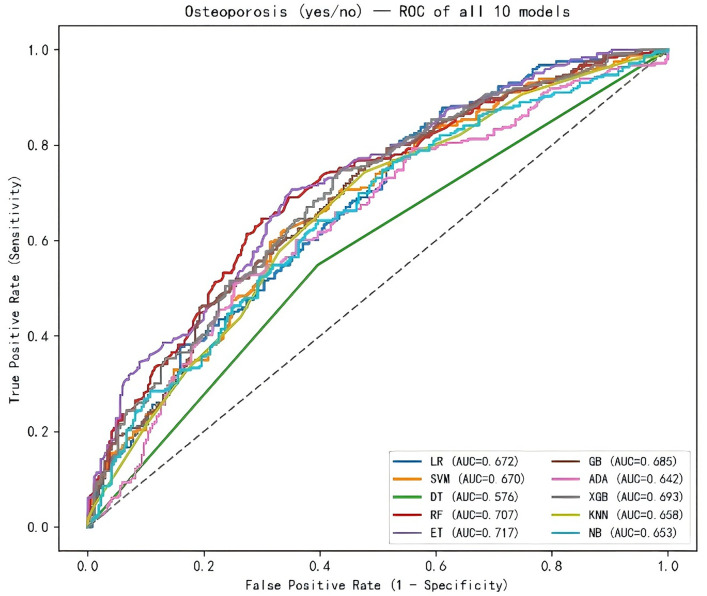
Comparison of the area under the receiver operating characteristic curves for 10 machine learning algorithms. Logistic Regression (LR, class_weight = balanced), SVM-RBF (SVM), Decision Tree (DT), Random Forest (RF), Extra Trees (ET), Gradient Boosting (GB), AdaBoost (ADA), XGBoost (XGB), KNN (K-Nearest Neighbors), Naive Bayes (NB).

With reference to the Recursive Feature Elimination (RFE) results from ten different algorithms (Fig 1), the assessment performance was optimal when all 30 clinical predictor variables were included. Therefore, all features were retained for model construction in this study. To mitigate the potential risks of overfitting and redundancy, we applied 10-fold cross-validation during training and subsequently used SHAP analysis to evaluate individual feature contributions, which helped identify the most influential predictors without prematurely removing variables that might collectively contribute to model performance. The initial performance of the ten models is presented in Table 4. In the preliminary evaluation stage, the AUC was designated as the primary metric for model assessment. Based on AUC values, three algorithms with superior performance were selected for further analysis: ET (AUC = 0.717), RF (AUC = 0.707), and XGB (AUC = 0.693), as illustrated in Fig 2.

**Table 4:** Comparison of the assessment results of each test model using test datasets.

Model	CV_AUC	AUC	ACC	Sens	Spec	PPV	NPV	F1
ET	0.727	0.717	0.647	0.589	0.700	0.642	0.652	0.614
RF	0.726	0.707	0.665	0.659	0.670	0.645	0.683	0.652
XGB	0.714	0.693	0.640	0.618	0.659	0.623	0.654	0.620
GB	0.708	0.685	0.626	0.614	0.637	0.606	0.644	0.610
LR	0.710	0.672	0.605	0.557	0.648	0.591	0.616	0.573
SVM	0.724	0.670	0.634	0.638	0.630	0.611	0.656	0.624
KNN	0.652	0.658	0.626	0.577	0.670	0.615	0.635	0.595
NB	0.664	0.653	0.607	0.48	0.722	0.611	0.604	0.538
ADA	0.664	0.642	0.614	0.602	0.626	0.594	0.633	0.598
DT	0.573	0.576	0.578	0.549	0.604	0.558	0.595	0.553

Logistic Regression (LR, class_weight = balanced), SVM-RBF (SVM), Decision Tree (DT), Random Forest (RF), Extra Trees (ET), Gradient Boosting (GB), AdaBoost (ADA), XGBoost (XGB), KNN (K-Nearest Neighbors), Naive Bayes (NB). AUCs with 95% confidence intervals (CIs) were computed using 2000 bootstrap replicates. The ET model achieved an AUC of 0.717 (95% CI: 0.682–0.752), while logistic regression achieved 0.672 (95% CI: 0.637–0.707). DeLong’s test showed a statistically significant difference between ET and LR (P = 0.023), though the absolute improvement was modest (ΔAUC = 0.045).

To further assess model robustness, AUCs with 95% confidence intervals (CIs) were calculated using 2000 bootstrap replicates. The ET model achieved an AUC of 0.717 (95% CI: 0.682–0.752), outperforming logistic regression (LR), which yielded an AUC of 0.672 (95% CI: 0.637–0.707). DeLong’s test demonstrated a statistically significant difference between ET and LR (P = 0.023), although the absolute improvement in discrimination was modest (ΔAUC = 0.045).

### Model development and selection

**Fig 3:**
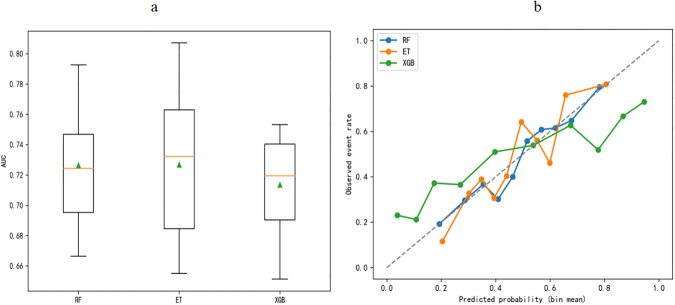
Comparison of the CV, AUC and predicted probability of RF, ET and XGB.

**Fig 4:**
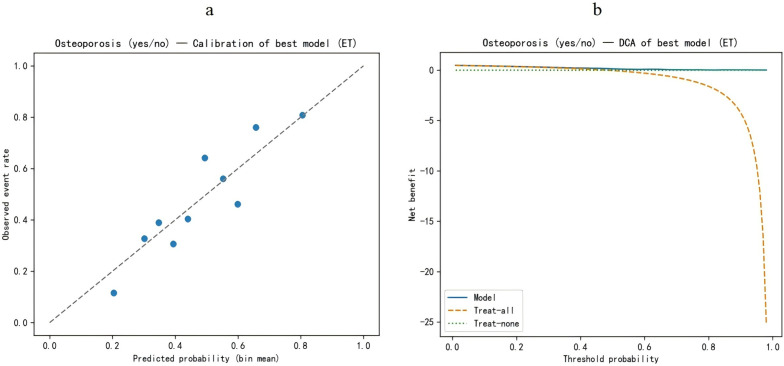
The best model calibration curve. **(a)** The decision curve of the best model. **(b)** The DCA of the model: Net benefit at 0.10 = 0.420, at 0.20 = 0.353, at 0.30 = 0.281, max net benefit = 0.471 at threshold 0.01.

**Fig 5:**
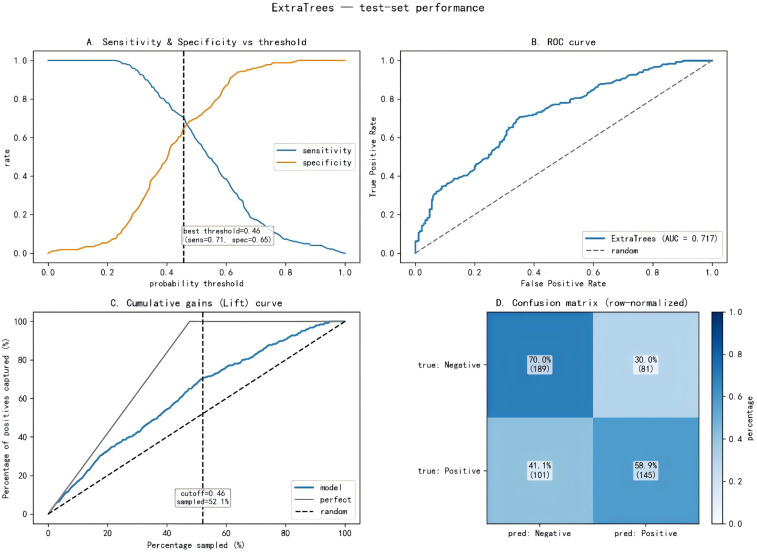
Extra Trees-test-set performance.

Based on the preliminary evaluation, further in-depth refinement was conducted on the three top-performing algorithms: ET, RF, and XGB. The hyperparameters of these algorithms were optimized using a randomized search method with 10-fold cross-validation. The optimized prediction models were then combined into a stacked ensemble model, which leverages the robustness of the ET, RF, and XGB algorithms synergistically. The ensemble was constructed using a two-stage approach: the first stage involved independently training the ET, RF, and XGB models on the designated training dataset, and their resulting associated factors were used as inputs to the second-stage model to generate an integrated assessment. To evaluate the reliability of the designed machine learning framework, its performance was benchmarked using 10-fold cross-validation on the training set. Therefore, the ET model was selected as the final model for subsequent SHAP analysis and interpretation. The AUC of 0.717 (95% CI: 0.682–0.752) indicates only modest discriminative ability, suggesting that further improvements in feature engineering or model architecture are needed. During internal validation, the ET model demonstrated better predictive performance than the standalone RF and XGB models, as shown in Figs 3 and 4. The calibration curves presented in Fig 5 provide insight into model calibration.

### Model performance and feature importance

**Fig 6:**
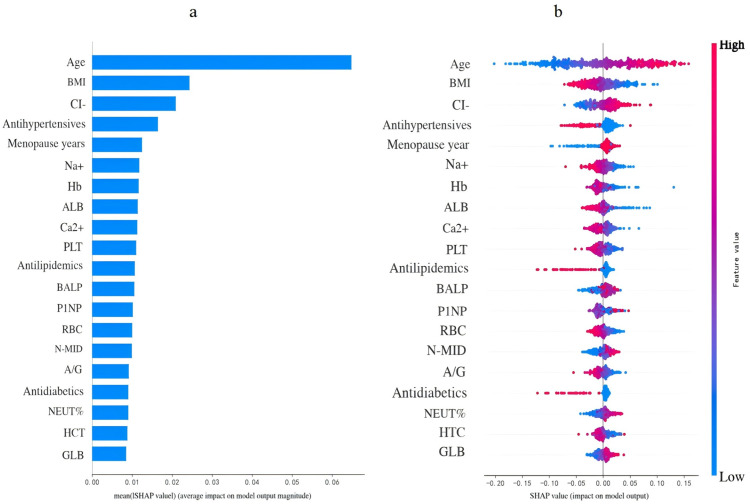
(a) Ranking of feature importance of the ET model based on SHAP values. Age: 0.0648; BMI: 0.0243; CI^-^:0.0209; Antipertensives: 0.0164; Menopause year: 0.0125; Na^+^: 0.0118; Hemoglobin: 0.0116; Albumin: 0.0114; Ca2^+^: 0.0113; PHT: 0.0110; Antilipidemics: 0.0107; BALP: 0.0105; P1NP: 0.0101; RBC: 0.0100; N-MID: 0.0099; A/G: 0.0092; Antidiabetics: 0.0090; NUET%:0.0090; Hematocrit: 0.0088; GLB: 0.0085. **(b)** Distribution of the impact of each feature on the output of the ET model estimated using SHAP values.

Although a stacked ensemble combining ET, RF, and XGB was constructed as described in the Methods, it did not yield improved performance over the standalone ET model in our cross-validation analysis. Therefore, the ET model was selected as the final model for subsequent SHAP analysis and interpretation. Although the ET model was selected as the best-performing algorithm, its AUC of 0.717 reflects only modest predictive discrimination, indicating that further improvements, such as incorporating additional predictive features or optimizing model architecture, are needed before clinical deployment. To better understand the impact of individual features on the ET algorithm-based OP risk assessment model, the SHAP values for each feature were calculated. Based on the ET model, the top 20 features were selected based on their importance ranking, as measured by mean absolute SHAP values (Fig 6a). The top five features, in descending order of importance based on mean absolute SHAP values, were age, BMI, chloride ion, use of antihypertensive medications, and years since menopause. Among bone turnover markers, bone-specific alkaline phosphatase (BALP) contributed the most, though only 0.0105, ranking 12^th^. The remaining bone turnover markers contributed relatively little. Fig 6b presents violin plots for each feature, illustrating the correlation between feature values and SHAP values. A larger absolute SHAP value for a feature indicates a greater influence of that feature on the ET-based prediction model. Red dots represent higher values of the feature, while blue dots represent lower values.

## Discussion

In this study, we developed and validated an explainable machine learning model to assess osteoporosis risk in postmenopausal women using routinely available clinical indicators. The ET algorithm demonstrated the best assessment performance with an AUC of 0.717, outperforming traditional methods such as logistic regression (ΔAUC = 0.045, P = 0.023). SHAP analysis identified age, BMI, and serum chloride as the most influential predictors, highlighting the multifactorial nature of postmenopausal OP and suggesting that electrolyte balance may influence bone health beyond traditional markers. These findings underscore the potential to integrate routine laboratory tests into osteoporosis risk assessment, thereby facilitating early identification of high-risk individuals in clinical practice. These findings are consistent with several recent studies. Yen et al. demonstrated that integrating imaging and clinical data using deep learning significantly improved fracture prediction accuracy (AUC = 0.88） [28], while Zitu et al. further confirmed that ML models exhibit superior generalizability in complex clinical settings compared to conventional statistical methods [29]. More specifically, our ET model is comparable to other clinical-feature-based ML models for OP. For instance, a study by Sun et al. [30] using only age and BMI achieved an AUC of 0.69 in a Korean cohort. The moderate performance of our model relative to Sun et al. may reflect the absence of specialized bone biomarkers in our routine clinical dataset. Conversely, our model outperformed simple logistic regression, consistent with previous reports that ensemble methods offer modest gains over traditional approaches.

Although our model demonstrated promising performance, with an AUC of 0.717, it did not reach the ideal threshold of 0.8, indicating room for improvement before clinical deployment. This moderate performance may be attributed to several factors, including data characteristics, model complexity, and evaluation metrics. First, the dataset was nearly balanced (47.7% OP vs. 52.3% non-OP), so class imbalance was not a major concern in this study. AUC is known to be robust to class imbalance when the class distributions are not extremely skewed. Thus, our evaluation metrics remain valid. Nevertheless, other factors, such as multicollinearity among the 31 clinical variables, may have affected model stability. Second, from a modeling perspective, while the ET algorithm, as an ensemble method, can handle high-dimensional features, the inclusion of 31 clinical variables, such as electrolyte levels and medication history, introduced potential multicollinearity issues. For instance, the notably large standard deviation of β-CTX may have adversely affected model stability. Finally, limitations inherent to the AUC metric must be considered. AUC only reflects the model’s ranking ability and does not account for the probability of calibration. For example, the calibration curve of the ET model in this study indicated discrepancies between assessment probabilities and actual outcomes (Fig 4a), suggesting suboptimal model fit. Moreover, AUC does not provide information on error distribution, potentially masking performance deficiencies in specific subgroups (e.g., advanced age or low-BMI populations) [31,32]. Therefore, future studies should incorporate more granular metrics (such as GAUC or F1-score) and external validation to comprehensively evaluate model performance.

This study further confirms the critical role of age in the development and progression of OP in postmenopausal women. SHAP analysis identified age as the most important associated factor (SHAP value: 0.0648), showing a significant positive correlation with OP risk. This finding is highly consistent with recent research on the role of cellular senescence in bone metabolism. With advancing age, senescent cells in the bone microenvironment secrete senescence-associated secretory phenotype factors (e.g., interleukins, metalloproteinases), creating a pro-inflammatory environment that activates osteoclasts and suppresses osteoblasts [33]. Senescent osteocytes also upregulate the RANKL/OPG ratio, promoting osteoclast differentiation [34]. Age-related mitochondrial dysfunction increases ROS production, inducing osteoblast apoptosis and promoting osteoclastogenesis via NF-κB activation.

Notably, we observed a negative correlation between BMI and OP risk (SHAP value: 0.0243), supporting the “obesity paradox”. Higher BMI may protect bone through mechanical loading (activating Wnt/β-catenin), aromatase-mediated estrogen production, and adipokine regulation (leptin, adiponectin) [35,36]. However, this protective effect is not linear, as extreme obesity may become detrimental due to chronic inflammation. The bidirectional relationship between BMI and OP observed in this study suggests that maintaining a moderate body weight may be beneficial for skeletal health in postmenopausal women.

In the current model, the significance of electrolyte indicators was identified through SHAP analysis, though their underlying mechanisms remain unclear. Notably, Cl⁻ ranked third among the most important features, after age and BMI, suggesting a potential role in bone metabolism. This may be attributed to the influence of electrolyte balance on the bone microenvironment, specifically by modulating pH or ion channels, which, in turn, affects osteoblast and osteoclast activity. Recent studies have revealed that ANO1 Cl^-^ channel plays a key regulatory role in osteoclast differentiation and bone resorption. ANO1 facilitates Cl^-^ efflux, enhancing H^+^ secretion and bone matrix dissolution. ANO1 also interacts with RANK, activating RANKL-RANK signaling and accelerating bone resorption [37]. Sodium ions influence calcium homeostasis via sodium-calcium exchange: high sodium intake increases urinary calcium excretion (20–60 mg Ca per 1000 mg Na), leading to negative calcium balance, and may inhibit intestinal calcium absorption while stimulating PTH secretion, thereby promoting bone loss [38]. However, these mechanisms were not fully captured in the multivariate model, likely due to data limitations and the model’s insufficient capacity to fit complex nonlinear relationships.

An important insight from our study is the relatively limited contribution of traditional bone turnover markers to the multivariate ML model. This finding does not negate the biological relevance of these markers but rather highlights that their predictive value may be overshadowed by more stable clinical features such as age and BMI when assessed in a multifactorial framework. From a clinical perspective, this suggests that while bone turnover markers remain valuable for monitoring treatment response, routine clinical parameters may be more practical for initial osteoporosis risk stratification in primary care settings. In our study, markers such as procollagen type I N-terminal propeptide (P1NP) and the N-terminal mid-fragment of osteocalcin (N-MID) exhibited relatively limited contributions to the prediction model. This aligns with the conclusion drawn by Yoo et al. in a Korean multicenter study, which demonstrated that the predictive value of individual BTMs is modest [39]. From a biological perspective, although traditional bone turnover markers reflect the rate of bone remodeling, their serum levels are subject to complex regulation by factors such as circadian rhythm and feeding status. Studies have shown that bone turnover markers exhibit significant circadian fluctuations, with variations ranging from 10% to 20% [40]. More importantly, postmenopausal osteoporosis involves a coupling imbalance between osteoblasts and osteoclasts within bone remodeling units, driven by immune cell-bone cell interactions (e.g., T-cell-secreted RANKL and Wnt signaling inhibitors) that are not captured by single biomarkers [41].

The 25(OH)D level did not show significant differences between OP and non-OP subjects, which may reflect its dual role in bone remodeling. While adequate vitamin D promotes bone mineralization, excessively high levels may stimulate bone resorption [42]. Our Northwest Chinese cohort is characterized by high latitude, limited sunlight exposure, and a wheat-based diet low in vitamin D-rich foods, potentially leading to vitamin D deficiency. This deficiency can trigger compensatory PTH elevation, accelerating bone turnover and obscuring a linear 25(OH)D-BMD association. A threshold effect exists, whereby the adverse effects of PTH on BMD become significant only when 25(OH)D falls below 20 ng/mL [43]. Additionally, VDR gene polymorphisms may modulate this relationship.

Given that our study cohort comprised exclusively postmenopausal women of Chinese (mainland) descent, the heterogeneity in ML-based osteoporosis prediction models across Asian populations warrants discussion. In Southern Taiwan, Huang et al. developed ML models using 2,638 health examination participants and found that artificial neural network achieved better performance than OSTA, with age, gender, and body weight as top predictors [44]. In contrast, our ET model identified age, BMI, and chloride as the most important features, suggesting that electrolyte markers may carry greater predictive value in mainland populations, possibly due to dietary or regional differences. Korean studies have also reported XGBoost models for osteoporosis risk classification in women, achieving accuracy of 0.705 and F1 of 0.738, with age at menopause as the strongest predictor [45]. Another Korean study incorporating genetic risk scores and up to 122 features achieved AUC exceeding 0.85 [46]. These cross-Asian comparisons highlight that model performance and feature importance vary substantially across populations, underscoring the need for region-specific model development and external validation before generalizing our findings to other Asian cohorts.

### Limitations

Although this study provides a machine learning perspective on risk assessment for OP in postmenopausal women, several limitations should be cautiously considered. First, because of the retrospective cross-sectional design, causal inferences cannot be made; the identified predictors should be interpreted as factors associated with PMOP rather than causal risk factors. Additionally, the data were sourced from only two tertiary hospitals in Northwest China, which may introduce selection bias and regional limitations, thereby limiting the model’s generalizability. Second, although multiple clinical and laboratory indicators were included, several potentially important predictors, such as detailed dietary patterns, alcohol intake, physical activity levels, sunlight exposure, genetic factors, and emerging biomarkers, were not incorporated, potentially limiting the predictive ability of the models. Furthermore, despite the use of cross-validation, the performance of the machine learning model (AUC = 0.717) remains moderate, indicating the need for further optimization in feature engineering and algorithm selection. Future studies should adopt prospective multi-center designs to enlarge sample size and improve geographical representativeness, and integrate multi-omics data (e.g., genomic, epigenomic, metabolomic) to develop more robust assessment models. In addition, external validation using independent cohorts from different regions and healthcare settings is strongly recommended to further evaluate the model’s stability, calibration, and clinical applicability, thereby enhancing its generalizability and translational value.

## Conclusion

This study developed and validated a machine learning-based assessment model for OP in postmenopausal women. The results demonstrated that the Extra Trees model exhibited the best predictive performance. SHAP analysis identified age, BMI, and electrolyte indicators, particularly Cl ⁻ , as the most influential clinical features associated with PMOP. Notably, the relatively limited contribution of traditional bone turnover markers in our multivariate model suggests that the pathophysiology of osteoporosis involves complex systemic interactions that may not be fully captured by individual biomarkers. This study presents an interpretable ML framework for assessing osteoporosis in postmenopausal women and highlights the value of integrating multidimensional clinical data. Future research should focus on prospective multi-center validation and the integration of emerging biomarkers to further enhance model generalizability and clinical utility.
